# Prokaryotic Argonaute Proteins: A New Frontier in Point-of-Care Viral Diagnostics

**DOI:** 10.3390/ijms241914987

**Published:** 2023-10-08

**Authors:** Kai Sun, Yan Liu, Wei Zhao, Biao Ma, Mingzhou Zhang, Xiaoping Yu, Zihong Ye

**Affiliations:** Zhejiang Provincial Key Laboratory of Biometrology and Inspection & Quarantine, College of Life Sciences, China Jiliang University, Hangzhou 310018, China; sunkai@cjlu.edu.cn (K.S.); ly1216331249@163.com (Y.L.); z15695530836@email.cn (W.Z.); 16a0701109@cjlu.edu.cn (B.M.); zmzcjlu@cjlu.edu.cn (M.Z.)

**Keywords:** viral detection, point-of-care diagnostics, prokaryotic argonaute, nucleic acid detection

## Abstract

The recent pandemic of SARS-CoV-2 has underscored the critical need for rapid and precise viral detection technologies. Point-of-care (POC) technologies, which offer immediate and accurate testing at or near the site of patient care, have become a cornerstone of modern medicine. Prokaryotic Argonaute proteins (pAgo), proficient in recognizing target RNA or DNA with complementary sequences, have emerged as potential game-changers. pAgo present several advantages over the currently popular CRISPR/Cas systems-based POC diagnostics, including the absence of a PAM sequence requirement, the use of shorter nucleic acid molecules as guides, and a smaller protein size. This review provides a comprehensive overview of pAgo protein detection platforms and critically assesses their potential in the field of viral POC diagnostics. The objective is to catalyze further research and innovation in pAgo nucleic acid detection and diagnostics, ultimately facilitating the creation of enhanced diagnostic tools for clinic viral infections in POC settings.

## 1. Introduction

Viruses, which are omnipresent infectious agents, can infect a diverse array of organisms globally. Recent epidemics, such as those caused by severe acute respiratory syndrome coronavirus 2 (SARS-CoV-2) [1], Zika virus (ZIKV) [2], and Ebola virus (EBOV) [3], have inflicted substantial socioeconomic damage. These outbreaks underscore the urgency for rapid, sensitive, and precise virus detection technologies, crucial for effective countermeasures against emerging and re-emerging viral threats.

Nucleic acid detection is pivotal in modern diagnostics due to its high sensitivity, specificity, and potential for swift results. Traditional methods like polymerase chain reaction (PCR) and reverse transcription polymerase chain reaction (RT-PCR) have revolutionized diagnostics by enabling the detection of specific pathogenic nucleic acid sequences. However, these methods often necessitate complex equipment, skilled personnel, and extended processing times, leading to a growing demand for faster, more accurate, and user-friendly diagnostic tools.

Point-of-care diagnostics, providing rapid, accessible, and accurate testing at or near the patient care site, are integral to contemporary healthcare [4]. These tools aim to minimize the time between testing and result acquisition, enabling timely informed decisions and interventions. The demand for POC diagnostics has surged due to the need for decentralized healthcare services, particularly in resource-limited settings and during infectious disease outbreaks. The development of novel nucleic acid detection platforms promises to enhance POC diagnostics.

Recently, Clustered Regularly Interspaced Short Palindromic Repeats-CRISPR associated (CRISPR-Cas) systems have emerged as potent diagnostic tools for nucleic acid detection, addressing some limitations of traditional methods. CRISPR-Cas13a-based SHERLOCK [5] and CRISPR-Cas12a-based DETECTR [6] technologies, demonstrating rapid, sensitive, and specific detection of various pathogens, including viral RNA and DNA, in POC settings, have become research focal points in viral molecular diagnostics. However, CRISPR-Cas detection technologies face challenges, including constraints imposed by the protospacer adjacent motif (PAM) or protospacer flanking sequence (PFS) on detection sequences and complications in multiplex target detection [7].

Simultaneously, Argonaute (Ago) proteins, ubiquitously conserved across various organisms, have demonstrated potential in nucleic acid detection [8]. These proteins, categorized into eukaryotic Argonaute (eAgo) and prokaryotic Argonaute (pAgo) based on their origin, can identify target RNA or DNA using complementary sequences via a single-stranded guide RNA or guide DNA. While the role of eAgo in the RNA interference (RNAi) pathway [9,10] is well-documented, pAgo, which exhibits diverse classifications and behaviors across different hosts, is garnering increasing attention. Certain Argonaute proteins have been identified to bind and cleave target DNA [11] and RNA [12] in a sequence-specific manner, and some pAgo proteins have demonstrated efficient cleavage of nucleic acids both in vivo and in vitro [8], broadening their potential applications.

pAgo proteins offer several advantages over Cas nucleases. Notably, pAgo proteins do not require the presence of Protospacer Adjacent Motif (PAM) sequences in target DNA, thereby providing more flexibility in the selection of nucleic acid targets [13]. Furthermore, most pAgo proteins employ short DNA molecules as guides, in contrast to Cas nucleases, which necessitate longer RNA guides. Given that DNA synthesis is more cost-effective and stable than RNA, this characteristic promotes the development of Ago-based nucleic acid detection systems. Additionally, pAgo molecules are smaller than Cas9, simplifying their molecular modification and production. Currently, the pAgo protein family is being utilized to develop high-sensitivity viral nucleic acid detection methods. Emerging Argonaute protein detection platforms leverage the inherent sequence-specific binding and cleavage properties of prokaryotic Argonaute proteins, offering a novel approach to detecting nucleic acids with high sensitivity and specificity.

This review aims to provide an in-depth exploration of the emerging Argonaute protein detection platforms and to critically evaluate their potential as competitors to CRISPR-based nucleic acid detection methods in the context of viral point-of-care (POC) diagnostics. By offering a comprehensive of Argonaute protein detection platforms, this review seeks to stimulate further research and innovation in the field of nucleic acid detection and diagnostics. The ultimate goal is to contribute to the development of more advanced diagnostic tools for viral infections and other diseases in POC settings.

## 2. Argonaute Proteins: Structure and Function 

### 2.1. Structural Overview

Argonaute proteins, a diverse family of nucleic acid-guided proteins, are ubiquitously found across various organisms [8]. Despite the low sequence homology between eAgo and pAgo, their fundamental structure and function remain highly conserved [13,14]. eAgo plays a crucial role in the RNA interference (RNAi) process, forming RNA-induced silencing complexes, binding to guide small single-stranded RNA molecules, and leveraging its inherent nuclease activity to either directly cleave target RNA or recruit other silencing proteins for target transcriptional repression [15].

**Figure 1:**
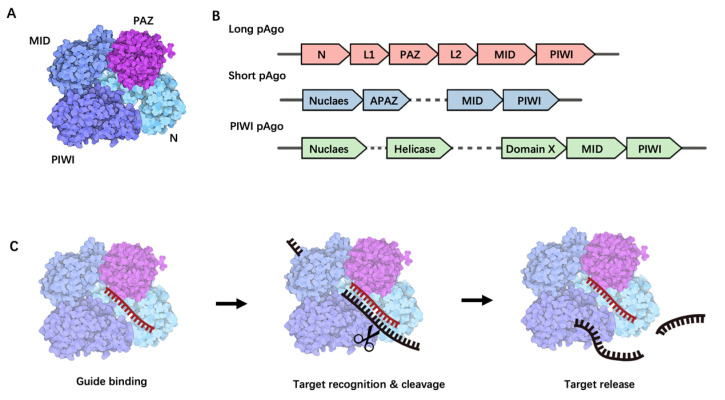
Structural and functional overview of Prokaryotic Argonaute Proteins. (**A**) The structural composition of long prokaryotic Argonaute proteins. (**B**) A schematic representation of long pAgo, short pAgo, and PIWI pAgo-encoding operon alongside a predicted protein scaffold, with individual genes denoted by separate arrows. (**C**) The process of nucleic acid cleavage by Argonaute proteins. This process begins with the loading of a guide single-stranded nucleic acid molecule onto Argonaute (guide binding), followed by a search for a complementary target. Upon finding a complementary target, the enzyme undergoes a conformational change, catalyzes the cleavage (target recognition and cleavage), and subsequently releases the cleaved target (target release).

pAgo, initially discovered in bacteria and archaea, can be classified into three structural categories: long pAgo, short pAgo, and PIWI-RE proteins [16]. Given that proteins used in nucleic acid detection predominantly belong to the pAgo family, this paper will primarily focus on reviewing the structure, function, and applications of pAgo proteins. Long pAgo is a well-studied class of Ago proteins, featuring structural domains highly reminiscent of eAgo, including N-terminal, PAZ (Piwi-Argonaute-Zwille), MID (middle), and PIWI (P-element Induced Wimpy Testis) domains (Figure 1A). The overall structure forms a bilobed scaffold, with one lobe comprising the N-PAZ domains and the other the MID-PIWI domains [15] (Figure 1A). The N-terminal domain, being the least conserved, may facilitate the separation of RNA duplexes. The PAZ domain, a small domain of approximately 140 amino acids, is involved in binding the 3′ end of the guide molecule. The MID domain houses a pocket for binding to the 5′ end of the guide. Upon target binding, the catalytically active PIWI domain mediates target strand cleavage. This domain contains a conserved tetra-amino acid residue DEDX (where X is an aspartate, histidine, or lysine residue), which can bind to the two divalent cations required for catalytic activity [15].

Short pAgo, in contrast, only consists of a MID domain and a PIWI domain, typically with an incomplete catalytic functional domain [17] (Figure 1B). Interestingly, genes encoding short pAgo proteins are often found adjacent to genes encoding analogs of Piwi-Argonaute-Zwille (APAZ) with unknown functions. It is hypothesized that short pAgo proteins may function as nucleic acid-guided target recognition platforms, whereas associated APAZ-nucleases may play a role in target nucleic acid degradation.

PIWI-RE proteins, similar to short pAgo, lack the N-terminal and PAZ domains but retain conserved MID and PIWI domains, suggesting that PIWI-RE proteins can provide structural support for nucleic acid-guided target recognition [18] (Figure 1B). Some PIWI-RE proteins also possess complete catalytic functional domains, indicating that a subset of these proteins may have the potential to cleave target nucleic acids [18]. PIWI-RE proteins are predominantly found in operons with DinG-type helicases and nucleases. It is postulated that helicases assist PIWI-RE proteins in accessing double-stranded nucleic acid targets, whereas nucleases contribute to guide generation and target nucleic acid cleavage [18].

### 2.2. Biological Functions of Long pAgo

pAgo proteins are hypothesized to play a significant role in cellular defense against viral invasion, a notion supported by recent studies on long pAgo proteins. Firstly, bioinformatics research reveals that Argonaute genes often co-locate in the same operon as genes encoding host defense proteins, such as restriction endonucleases [19]. Secondly, some prokaryotic Argonautes contain Silent Information Regulator 2 (SIR2) or Toll/interleukin-1 receptor (TIR) domains, either within the protein or in neighboring genes [17]. These domains are typically part of the bacterial anti-phage Thoeris defense mechanism [20,21]. Recent experimental evidence has demonstrated that long pAgos participate in bacterial immune defense responses, such as CbAgo (from *Clostridium butyricum*) protecting cells from phage invasion [11], and TtAgo (from *Thermus thermophilus*), PfAgo (from *Pyrococcus furiosus*) [22] and RsAgo (*Rhodobacter sphaeroides*) [23] reducing plasmid transformation efficiency, thereby promoting the degradation of exogenous plasmids in bacterial cells. Beyond protecting cells from foreign nucleic acids, Argonautes can perform other functions. For instance, TtAgo binds 15–18 nt DNA fragments from DNA replication termini and interacts with DNA replication-related proteins, thereby participating in the completion of DNA replication [24]. The mechanisms of TtAgo and PfAgo in host defense responses have been extensively studied, with the specific mechanisms described below.

TtAgo, derived from *Thermus thermophilus* [25], reduces plasmid transformation efficiency and is presumed to serve as a defense against foreign nucleic acid invasion. Further research shows that TtAgo mediates DNA-guided DNA interference, directly cleaving and invading foreign nucleic acids in bacteria at the DNA level [26]. In vivo, TtAgo forms a complex with small interference DNA (siDNA) guide molecules (13–25 nucleotides in length) and directs single-stranded target DNA binding and cleavage. In some cases, two complementary TtAgo-siDNA complexes can introduce dsDNA breaks [25] via which TtAgo degrades invading DNA and reduces the levels of plasmids in cells. In vitro, TtAgo cleaves the phosphodiester bond between the 10th and 11th bases of the target DNA from the 5′ end using a 5′-phosphorylated ssDNA as the guide DNA (gDNA). Studies have shown that the minimum length of gDNA is as short as 9 nt [25], and it does not depend on a PAM sequence. Moreover, TtAgo has the ability to cleave RNA in vitro and can also reduce the levels of RNA transcribed from invading DNA in vivo [25]. However, whether this decrease in RNA levels is a direct result of RNA-directed degradation or an indirect result of DNA interference remains to be proven.

PfAgo, derived from *Pyrococcus furiosus*, exhibits a higher reaction temperature range (87 °C to 99.9 °C) compared to TtAgo. Similar to other bacterial-derived pAgos, the archaeal PfAgo protein reduces the plasmid transformation efficiency of *Pyrococcus furiosus*, suggesting a role for PfAgo in defending against foreign nucleic acid invasion [22]. Studies on PfAgo’s in vitro cleavage activity reveal that PfAgo can bind to 5′-phosphorylated ssDNA (gDNA) at high temperatures and direct the cleavage of complementary single-stranded DNA. The results suggest that gDNA within the length range of 15–31 nt better mediates PfAgo’s cleavage of single-stranded DNA (ssDNA). Behnam Enghiad et al. [27] discovered that under high-temperature reaction conditions, providing paired gDNA can mediate PfAgo to cleave double-stranded DNA (dsDNA) and produce specific termini. The PfAgo-based nucleic acid cleavage system can recognize any region of DNA and has strong designability and operability. It has been applied to dsDNA-targeted enzymatic cleavage and rapid detection of pathogenic microorganisms and tumor marker genes.

Other long pAgos include AfAgo (from *Archaeoglobus fulgidus*), AaAgo (from *Aquifex aeolicus*), MpAgo (from *Marinitoga piezophila*), and TpAgo (from *Thermotoga profunda*). In vitro, AfAgo prefers ssDNA as the guide nucleic acid sequence to bind with target DNA, whereas AaAgo is involved in DNA-mediated RNA cleavage [19]. Genes encoding MpAgo and TpAgo are often found in the same gene cluster as those encoding CRISPR-Cas enzymes, suggesting functional links between the two [28]. Unlike most pAgos that utilize 5′-phosphorylated gDNA, MpAgo and TpAgo use 5′-hydroxylated gRNA as guides to cleave specific ssDNA [28]. Moreover, MpAgo can also use 5′-hydroxylated RNA guides to cleave target ssRNA [28], suggesting that although the roles of MpAgo and TpAgo may be similar to other long pAgos, their mechanisms for generating and binding guide molecules are different [28]. Bioinformatics analysis suggests that some genes encoding long pAgos are fused with genes encoding Schlafen-like ATPases, whereas others co-occur in operons with genes encoding Mrr, Sir2, Cas4-like, or Phospholipase D (PLD) nucleases [17]. It is expected that these pAgo-associated enzymes are involved in processes such as guide nucleic acid generation, target DNA unwinding, or degradation. There is a growing number of studies revealing the functions and properties of different types of pAGO (Table 1). 

## 3. pAgo-Based POC Detection Technologies

**Figure 2:**
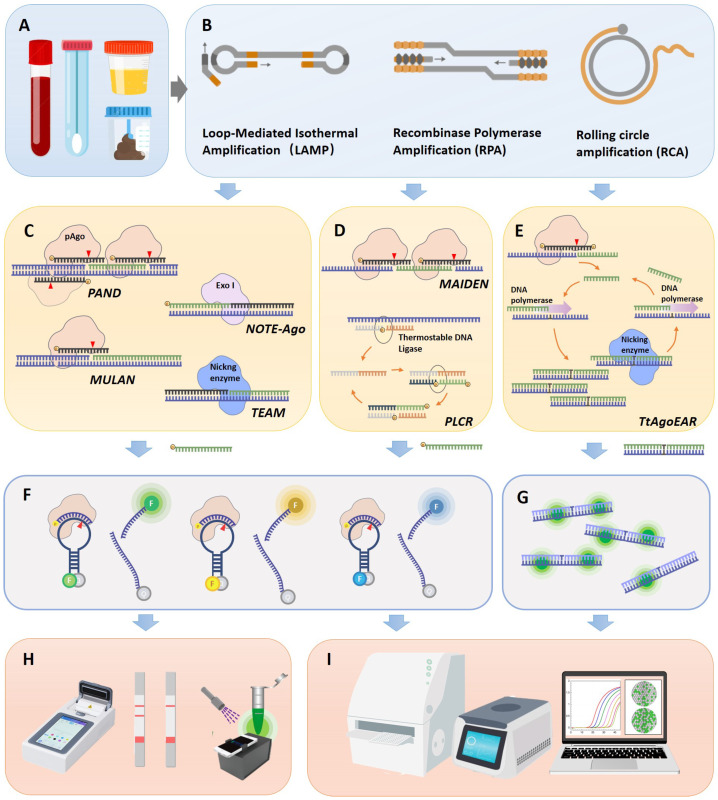
Workflow of pAgo-based nucleic acid detection system for viral diagnosis. (**A**) The collection of clinical samples from patients, including blood, throat swabs, urine, and fecal samples. (**B**) The enrichment of viral nucleic acid target sequences, potentially employing isothermal nucleic acid amplification techniques such as LAMP, RPA, and RCA. (**C**) The working principle of pAgo nucleic acid detection systems for double-stranded DNA target sequences, showcasing PAND, NOTE-Ago, MULAN, and TEAM detection platforms. (**D**) The working principle of pAgo nucleic acid detection systems for single-stranded DNA target sequences, exemplifying MAIDEN and PfAgo coupled with modified ligase chain reaction for nucleic acid detection (PLCR) detection platforms. (**E**) The working principle of pAgo nucleic acid detection systems for RNA targets, featuring the TtAogEAR detection platform. (**F**) The detection of fluorescence probes. The probe was designed as an ssDNA sequence complementary to the newly generated Gdna. It is accompanied by a fluorophore (represented by different colored ’F’ markers in the figure, which correspond to various fluorophores such as carboxyfluorescein (FAM), Victoria (VIC), or rhodamine-X (ROX)). On the other end, the gray ’q’ symbolizes a quencher (for instance, black hole quencher-1 or 2 (BHQ1 or -2)). (**G**) The detection of SYBR Green fluorescence signals. (**H**) Portable fluorescence signal reading devices, including integrated isothermal amplification detection devices, lateral flow test strips, and 3D-printed fluorescent readers. (**I**) Laboratory fluorescence detection devices, such as enzyme-linked immunosorbent assay (ELISA) readers, qPCR machines, or digital PCR machines.

The utilization of pAgos for viral nucleic acid detection encompasses a series of steps: Initially, viral nucleic acid was extracted from clinical samples and the target (either DNA or RNA) undergoes amplification via PCR or isothermal amplification, with RNA viruses necessitating an extra reverse transcription step (Figure 2A,B). Following this, a 5′ phosphorylated guide nucleic acid is designed to target the sequence, directing pAGO to execute the first round of cleavage on the target DNA amplicon (Figure 2C–E). This process yields secondary 5′ phosphorylated guide nucleic acids originating from the target sequence. These 5′ phosphorylated terminal DNA fragments act as new guide strands to cleave fluorophore-quencher labeled probes (Figure 2F), which can subsequently be detected via fluorescence spectrophotometry, quantitative real-time PCR (qPCR), droplet digital PCR (ddPCR), or a microplate reader (Figure 2H,I). Notably, pAGO, devoid of trans-cleavage activity, can cleave multiple targets and corresponding probes by designing several guide strands, thereby facilitating multiplex targeting of viruses [40,41,42]. Additionally, the cleavage activity of Agos can enrich mutant alleles by directly degrading wild-type DNA or RNA targets, a feature that can be harnessed for viral typing diagnosis [43]. Moreover, certain studies employ exponential amplification reaction (EXPAR) [42,44] or Ligase chain reaction (LCR) technology [40] to guide the generation of guide nucleic acids complementary to the target sequence, serving as new guide strands, directing pAGO to cleave fluorophore-quencher labeled probes. 

Viral detection systems predicated on pAgo proteins have been developed for a range of viruses, including SARS-CoV-2, Middle East Respiratory Syndrome (MERS-CoV), SARS-CoV, Human Papilloma Virus (HPV), and influenza virus, underscoring the expansive application potential of this technology. It is noteworthy that a multitude of Point-of-Care testing (POCT) equipment development and application studies exist for isothermal amplification and fluorescence probe detection. Such devices, encompassing portable fluorescence analyzers, test strips, and microfluidic products, can be swiftly adapted to pAGO-based detection technology, facilitating expedited processing from sample collection to result interpretation. In the subsequent section, we will delve into a comprehensive analysis of the principles and characteristics of various types of pAGO detection methods (Table 2).

### 3.1. PfAgo-Mediated Nucleic Acid Detection, PAND

PAND is a PfAgo-based nucleic acid detection method [45]. He et al. found that when target dsDNA (dsDNA1) is used as a substrate, the PfAgo-DNA complex can specifically cleave it and generate new short ssDNA. In the same reaction system, the newly generated short ssDNA can bind to PfAgo protein, which can further initiate the second round of cleavage of downstream fluorescent probes, producing a fluorescent signal as an indicator of the presence of the target nucleic acid [45]. He et al. successfully conducted a simultaneous five-channel detection of HPV11, HPV16, HPV18, HPV33, and HPV45 subtypes in clinical serum samples. They utilized molecular beacons labeled with five non-fluorescent molecules (HEX, CY5, 6-FAM, CY3, and TET) and extracted DNA as the testing subject. And the minimum detectable concentration of DNA was 160 fM. Furthermore, a sensitivity of 1.6 aM was achieved when PAND was coupled with a nucleic-acid amplification step [45]. Wang et al. established a detection method for COVID-19 based on PAND technology [46], using viral RNA from nasopharyngeal or oropharyngeal swabs, and conserved regions of the viral genome were amplified via RT-PCR. The resulting PCR products were combined with PfAgo, guide DNAs, and molecular beacons. Following a 20–30 min incubation at 95 °C, fluorescence signals were detected. This method reduced the entire detection process to about an hour, and the real-time fluorescence quantitative PCR instrument only needed for 3–5 min per batch, lessening the reliance on expensive Real-time PCR devices. The platform’s precision, due to gDNA nucleotides, facilitated the detection of the SARS-CoV-2 D614G variant. Clinical tests using SARS-CoV-2 PAND aligned perfectly with RT-qPCR outcomes. In comparison to CRISPR technology, PAND technology manifests paramount advantages, exemplifying features such as flexibility in PAM sequence, increased DNA-guided stability, and the capability for quintuple-channel multiplex detection utilizing a singular Ago protein. Additionally, it harbors substantial prospects for marketization in diagnostic apparatuses. For example, XUN et al. have meticulously described a Scalable and Portable Testing (SPOT) system, integrated with a battery-powered portable device, specifically engineered for the detection and diagnosis of COVID-19 [47]. The SPOT assay amalgamates a singular pot reverse transcriptase-loop-mediated isothermal amplification (RT-LAMP), subsequently followed by PfAgo-based target sequence discernment. This assay is characterized by its rapidity, with a detection time of less than 30 min, and high sensitivity, detecting the N gene and E gene in a multiplexed reaction with the limit of detection (LoD) of 0.44 copies/μL and 1.09 copies/μL, respectively. Moreover, the assay demonstrates exceptional precision, exhibiting 93.3% sensitivity and 98.6% specificity. Such attributes of speed, sensitivity, and accuracy are pivotal, enabling extensive, cost-effective access to areas in dire need of COVID-19 testing resources.

### 3.2. Ultra-Short PCR and Pyrococcus furiosus Argonaute Combined Nucleic Acid Detection (USPCRP)

He et al. devised a USPCRP nucleic acid detection system that amalgamates ultra-short PCR (usPCR) amplification technology with the PfAgo cleavage system [48]. This approach does not necessitate input gDNA but rather employs two primers shorter than 14 nt (one modified with a 5′ phosphate group) to amplify target DNA via ultra-short PCR. This results in a DNA product with a 5′ phosphate group modification, which serves as gDNA for the directed cleavage of fluorescent probes. Given that PfAgo is insensitive to gDNA shorter than 14 nt, the amplification primers with 5′ phosphate group modification do not induce nonspecific cleavage of the fluorescent probes. The USPCRP system was utilized to detect the ORF1ab gene of SARS-CoV-2, MERS-CoV, and SARS-CoV, demonstrating high sensitivity (10 aM) and high specificity single-base resolution nucleic acid detection, and successfully identifying the virus in clinical samples acquired from nasopharyngeal or oropharyngeal swabs, exhibiting a high level of concordance with the outcomes obtained via RT-qPCR. The system generates functional gDNA using usPCR, significantly reducing target fragment enrichment time. The paramount innovation of this system, when contrasted with analogous technologies, resides in its freedom from PAM constraints, necessitating merely two enzymes (Taq DNA polymerase and PfAgo), and its amplification of the detection system’s operability. Nonetheless, it bears multiple limitations during its application, which could potentially hinder its commercialization trajectory. It is vital to recognize that, due to the concise length of usPCR products, the choice of a DNA polymerase devoid of 3′–5′ exonuclease activity, such as Taq DNA polymerase, is essential to avoid gDNA degradation. This denotes a heightened likelihood of introducing mutations during the amplification phase, constraining its utility in SNP detection. Furthermore, this technique faces challenges in realizing multi-target detection, as the integration of short primers may escalate the probability of nonspecific amplification.

### 3.3. Ago-Directed Specific Target Enrichment and Detection (A-Star)

A-Star is an ultra-sensitive, single-tube, single-nucleotide mutation enrichment detection system [49]. This system incorporates mismatched and phosphorylated gDNA into the PCR reaction system, directing PfAgo to specifically cleave wild-type sequences during the denaturation step of PCR. This process rapidly enriches rare mutant alleles in the subsequent amplification step, with the amplified products then being suitable for various downstream detection methods, including Sanger sequencing and TaqMan quantitative real-time PCR. Compared to traditional methods, A-Star significantly enhances the sensitivity of detecting rare variant alleles, capable of specifically amplifying Single nucleotide variants (SNV) alleles as low as 0.01%, with an enrichment efficiency exceeding 5500 times. For instance, the researchers evaluated A-Star using DNA extracted from tissue (33.3 ng of genomic DNA) and blood samples (3.3 ng of cfDNA) of patients with various cancers harboring the Kirsten ras oncogene G12D Single Nucleotide Variation (SNV). The results indicated successful enrichment and detection of SNVs by A-Star. The variant allele fractions in tissue samples processed directly by A-Star surged to 60–90%, up from the initial VAFs of less than 20%. Given its attributes, this technique is set to emerge as a swift, economical, and highly sensitive method for rare SNV detection, holding promise for broad applications in cancer and viral variant identification. 

### 3.4. Multiplex Ago-Based Nucleic Acid Detection System (MULAN)

Ye et al. developed a multi-target detection platform, MULAN, by integrating RT-LAMP isothermal amplification technology with the PfAgo cleavage system [43]. This platform consolidates the nucleic acid detection process into a single-tube system, facilitating multiplex nucleic acid detection of different fluorophores via portable devices or qPCR instruments. Using a meticulous design of gDNA and reporter probes, the authors employed this novel method to swiftly identify SARS-CoV-2 WT and its D614G variant. The detection time for RT-LAMP is 35 min, and PfAgo cleavage takes 15 min, with a limit of detection (LoD) for each reaction as low as five copies, surpassing the Cas13a-based SHERLOCK detection system (LoD of 42 copies per reaction within 60 min). Clinically validated, MULAN is capable of swiftly conducting triplex detection of SARS-CoV-2, influenza A, and influenza B viruses in one reaction. This was achieved using nasopharyngeal swabs from patients with confirmed influenza viruses and Pseudoviruses representing SARS-CoV-2. Notably, MULAN’s results align perfectly, showing 100% agreement with RT-PCR outcomes. To achieve visualization and reduce dependence on detection instruments, the authors also developed a paper-based detection method with a limit of detection of 15 copies per reaction. The principal advantage of integrating isothermal amplification with the PfAgo platform via MULAN lies in its resolution of the low specificity issue inherent in isothermal amplification. Additionally, it maintains simplicity in design and implementation for multiplex detection, in contrast to the currently advanced CRISPR-based tests that necessitate multiple enzymes. Furthermore, the detection workflow designed by MULAN is meticulously optimized for POCT, integrating two sequential reactions in designated chambers. This allows the reaction to transpire within a single tube without the necessity for lid-opening operations, thereby mitigating contamination risks. Concurrently, the reaction tube is compatible with portable fluorescence detectors, enhancing its marketability. The portable implementation of MULAN offers an economical and user-friendly solution for detecting multiple pathogens, a requirement paramount for managing infectious diseases.

### 3.5. TtAgo-Assisted Exponential Isothermal Amplification for Multiplex Detection (TEAM)

TEAM is a multiplex nucleic acid detection system based on TtAgo that merges programmable TtAgo cleavage with exponential amplification reaction (EXPAR) for multiplex detection [41]. While traditional EXPAR provides excellent amplification efficiency, its sensitivity is limited due to non-specific background signals. By combining Ago’s programmability, precise specificity, and multi-round cleavage activity with EXPAR’s efficient amplification, TEAM achieves single nucleotide discrimination and high sensitivity down to single-molecule concentrations. TtAgo significantly accelerates the detection process, reporting the presence of miRNA in just 10–15 min, reducing the total time for miRNA detection to approximately 30–35 min. By incorporating different fluorescently labeled probes into the detection system, the TEAM system supports the simultaneous detection of four groups of target nucleic acids. The performance of the TEAM assay in diagnosing colorectal cancer (CRC) was evaluated by concurrently detecting several circulating miRNAs in clinical serum samples. Through the use of the TEAM system, a more pronounced fluorescence was noted in the cancer cohort compared to the normal cohort. This suggests elevated levels of targeted miRNAs, specifically miRNA-21, miRNA-92a, miRNA-31, and miRNA-141, in patients with CRC. The statistical significance, as indicated by the *p*-values, underscores the exceptional diagnostic capability of the TEAM method for CRC. Notably, this outcome surpasses the results obtained solely using EXPAR and RT-PCR analyses. Due to TtAgo’s single nucleotide accuracy, the system holds great potential for multiple-target and multi-type classification detection of viruses, with potential applications in the field of virus detection.

### 3.6. Mesophilic Ago-Based Isothermal Detection Method (MAIDEN)

Li et al. ingeniously tackled the challenge of the high-temperature requirement inherent in TtAgo or PfAgo-based methods [29]. They achieved this by broadening their research scope to include mesophilic Agos, thereby facilitating isothermal detection of the CRISPR-like mechanism. This novel approach, termed Mesophilic Ago-Based Isothermal Detection Method (MAIDEN), integrates mesophilic Ago cleavage with reverse transcription. This combination yields single-strand DNA as a substrate and facilitates the cleavage of fluorescence probes, enabling the detection of in vitro transcribed SARS-CoV-2 RNA at moderate temperatures. The initial phase involved mining and optimizing the mesophilic Ago and the fluorescence reporter system, followed by identifying a compatible reverse transcription reaction. Subsequently, they streamlined MAIDEN into a one-step process capable of detecting SARS-CoV-2 RNA at nanomolar concentrations, maintaining a steady temperature of 42 °C within an hour. This refined approach offers portability and ease of operation while reducing the risk of contamination from open-lid scenarios. This pioneering study illustrates the potential of mesophilic Agos as diagnostic tools and suggests that MAIDEN could be adapted to rapidly and efficiently detect a variety of pathogens. Regrettably, this methodology has not been applied to clinical sample testing, hence the exploration and research into its practical application and marketability remain pending.

### 3.7. Tt Argonaute-Based Thermostable Exponential Amplification Reaction (TtAgoEAR)

In an effort to consolidate the pAgo-mediated cleavage step and the amplification step into a singular isothermal reaction for RNA analysis, Yuan et al. developed an innovative isothermal amplification strategy. This approach, named *Thermus thermophilus* Argonaute-based Thermostable Exponential Amplification Reaction (TtAgoEAR) [44], enables RNA detection with ultra-sensitivity and single-nucleotide resolution at a constant temperature of 66 °C. The TtAgoEAR system operates using two sequential circuits. The first circuit is designed to derive the desired single-stranded initial oligonucleotide from an RNA target. A 16-nt gDNA, complementary to the target RNA and phosphorylated at both the 5′ and 3′ ends, is designed to activate Ago proteins while inhibiting its extension. The gDNA and TtAgo form a complex that specifically recognizes RNA and generates a nick, leading to the formation of the initial oligonucleotide. The second circuit’s objective is to amplify the initial oligonucleotide released from the first circuit using a single template. This template comprises a central nicking enzyme recognition site and a repetitive sequence complementary to the trigger at both ends. Upon the addition of the target RNA, the produced initial oligonucleotide hybridizes to the template and is extended to form double-stranded DNA by a DNA polymerase. The cleavage of double-stranded DNA by a nicking enzyme generates a new trigger sequence that initiates the repeated cycle of DNA replication via a cycle of hybridization, elongation, and cutting reactions. The exponential reaction process of TtAgoEAR can be monitored in real time using the dye SYBR Green I. TtAgoEAR enables the detection of different types of RNA, showcasing the effectiveness of the coupled TtAgo cleavage and thermostable EXPAR in the TtAgoEAR method. The research team assessed the efficacy of the TtAgoEAR analysis by testing 12 clinical saliva samples. When compared to qRT-PCR results, the TtAgoEAR method accurately identified three of the four SARS-CoV-2 positive samples and correctly recognized all the negative samples. This suggests that the proposed method possesses a reliable capability to detect SARS-CoV-2 infections in patients. Moreover, the amalgamation of TtAgoEAR with lateral-flow-based readouts enables the portable examination of samples at the point of care, presenting substantial prospects for commercialization.

### 3.8. Short Prokaryotic Argonaute/TIR-APAZ (SPARTA)-Based Nucleic Acid Detection Tool

This article primarily elucidates the application of long Ago proteins in the realm of viral nucleic acid detection while also recognizing the potential utility of short Ago proteins in nucleic acid detection. For instance, Koopal B and colleagues delineated the functionality and mechanism of a novel short prokaryotic Argonaute/TIR-APAZ (SPARTA) defense system [50]. The SPARTA systems are structured with a catalytically inactive short pAgo and a TIR-APAZ protein, together forming a heterodimeric complex. In this arrangement, the short pAgo operates as the ‘sensor,’ employing guide RNAs to attach to single-stranded (ss) DNA targets. Upon the binding of a target, the NAD(P)ase activity of the TIR-APAZ ‘effector’ becomes unleashed. In vivo, SPARTA targets invading plasmid DNA, causing a reduction in the cellular levels of metabolites NAD+ and NADP+, subsequently leading to cell death.

In the application of SPARTA for ssDNA detection, synthetic RNA guides complementary to ssDNA, along with ɛ-NAD (an analogue of NAD+), are introduced into the system. When the target ssDNA is present, SPARTA is activated to form a complex, which subsequently converts ɛ-NAD into fluorescent ɛ-ADPR. SPARTA can also be modified to detect dsDNA, enhancing the sensitivity of SPARTA-based detection. Target DNA can be specifically amplified via PCR using a phosphorothioate (PT) forward primer and an unmodified reverse primer. After incubation with T7 exonuclease, the unmodified strand is degraded, leaving behind ssDNA fragments containing the target sequence detectable by SPARTA. While the detection methods based on SPARTA are still in the conceptual phase, they pave the way for potential point-of-care (POCT) applications of this technology. For instance, SPARTA can be integrated with isothermal amplification techniques, and the fluorescent signal generated in the presence of target sequences can be detected using handheld devices offering a promising avenue for more accessible and specific diagnostic applications [51].

**Table 2:** Comparison of different Ago-based detection platforms.

Detection Platform	pAgo Protein	Amplification	Limit of Detection (LoD)	Target/Type	Detection Time	Signal Reading	Features	Reference
PAND	PfAgo	PCR or tHDA	1 copy/μL	HPV/DNA	2 h	qPCR system	Multiplex target detection short qPCR instrument usage time	[45]
USPCRP	PfAgo	usPCR	10 aM	SARS-CoV-2, MERS-CoV, SARS-CoV/RNA	70 min	Fluorescence detector ^1^	less than two enzymes include short target enrichment time, detection of extremely short targets	[48]
A-Star	PfAgo	PCR or RT-PCR	0.01% mutant	KRAS G12D/DNA	N/A	qPCR system, Sanger sequencing	Improve the amplification efficiency of mutant genes,	[49]
MULAN	PfAgo	RT-LAMP	5 copies/μL	SARS-CoV-2, influenza virus/RNA	35 min	Blue-light, qPCR system Lateral flow dipstick	One-pot detection, multiple detection	[43]
TEAM	TtAgo	EXPAR	1 aM	let-7/miRNA	35 min	Fluorescence detector ^1^	Multiple miRNA detection	[41]
TtAgoEAR	TtAgo	EXPAR	20 aM	SARS-CoV-2, HOTTIP/RNA	80 min	qPCR system	Adaptable to a lateral-flow-based readout	[44]
PLCR	PfAgo	LCR/RT-LCR	1 aM	HPV, SARS-CoV-2/DNA or RNA	100 min	fluorescence plate reader	Multiple detection	[40]
NOTE-Ago	PfAgo	PCR	1 CFU/mL	Salmonella typh, Staphylococcus aureus/DNA	2 h	3D-printed fluorescent reader	Fluorescent visualization	[52]
RADAR	PfAgo	PCR or RT-PCR	10–15 M	HPV, DNA	2 h	Fuji FLA7000 scanner	Multiple detection, gene genotyping	[37]
MAIDEN	Mesophilic Ago	Reverse transcription	1 nM	SARS-CoV-2, RNA	60 min	qPCR system	Portable, single-tube detection	[29]

^1^ The article does not indicate the name and model of the specific testing instrument.

## 4. Discussion

Nucleic acid detection technologies, such as PCR, RT-PCR, Isothermal Amplification, and CRISPR-Cas systems, are integral in the field of virus diagnostic [53,54]. The selection of an optimal technology for POC is contingent upon specific diagnostic prerequisites, including sensitivity, specificity, and rapidity, necessitating the development of portable, user-friendly, and cost-effective POC tests to enhance diagnostic accessibility in resource-constrained settings.

Each technology presents distinct advantages and limitations. PCR and RT-PCR, recognized as the gold standards for viral nucleic acid detection, offer high sensitivity and specificity, but their requirement for sophisticated equipment and thermal cycling renders them time-consuming and susceptible to contamination, necessitating skilled operation [54]. Isothermal Amplification is notable for its speed and suitability for POC testing in resource-limited settings, eliminating the need for thermal cycling, and simplifying equipment requirements. It can integrate with lateral flow assays for visual readout, though it may compromise specificity and face challenges with limited multiplexing capability and contamination susceptibility [55].

CRISPR-Cas technology, renowned for its versatility and high specificity, can target any DNA/RNA sequence, making it a promising candidate for POC applications. Recent advancements have developed CRISPR-based nucleic acid detection technologies that do not rely on nucleic acid amplification and POCs utilizing microfluidic technology [56]. However, it faces challenges, including off-target effects, constraints imposed by the PAM or PFS on detection sequences, and complications in multiplex target detection.

Argonaute (Ago)-based nucleic acid biosensors, when juxtaposed with traditional detection techniques, offer a multitude of advantages, thereby positioning them as a promising platform for the next generation of nucleic acid biosensing. One of the key advantages of Argonaute is its lack of a PAM sequence requirement in target DNA, which provides greater flexibility in the selection of nucleic acid targets. Furthermore, the guide molecules of the majority of prokaryotic Argonautes are short DNA molecules [16]. Their high stability, simplicity, and lower production costs make them ideal for the creation of cost-effective, easy-to-store reagents for point-of-care testing (POCT) applications, thereby streamlining both laboratory and field-testing procedures. The high specificity between guide DNA (gDNA) or guide RNA (gRNA) and target nucleic acids facilitates the detection of single-base mutations in viruses. Additionally, compared to other in situ detection technologies, Ago-based biosensors are more adept at achieving multiplex nucleic acid detection [37,40,57].

However, there are also challenges associated with Ago-based biosensors. Unlike the self-signal amplification mechanism inherent in CRISPR-Cas, Ago nucleic acid detection necessitates additional amplification or enrichment of target nucleic acids to bolster detection sensitivity. Given that Ago cleavage is directional, its efficiency is not as high as the trans-cleavage of Cas proteins. Augmenting the cleavage efficiency of Ago could potentially facilitate signal amplification, potentially enabling the realization of pAGO detection technologies that do not rely on nucleic acid amplification. but this would likely require intricate sequence design and could introduce additional enzymes and nucleic acid sequences, thereby increasing the complexity of the application [40,44]. Furthermore, the design of Ago-based biosensors must consider the tolerance of gDNA or gRNA mismatches at specific nucleotide positions. While PfAgo and TtAgo are predominantly used for in vitro detection, the in vitro application of other Agos is limited, and the exploration of most Agos in vitro remains a work in progress.

In domains extending beyond the scope of viral nucleic acid detection, pAgos are broadening their applicative reach, venturing into areas such as super-resolution microscopy and the meticulous visualization of miRNA, utilizing advanced methodologies including DNA-PAINT and Ago-FISH [57,58]. Argonautes are surfacing as prospective contenders for innovations in genome and transcriptome editing [16]. They possess the capability to modulate gene expression by recruiting epigenetic modifying factors and can be strategically amalgamated with a variety of domains and proteins to cater to a spectrum of applications, spanning from intricate visualization to the post-transcriptional regulation of gene expression. The comprehensive and multifaceted capabilities of Argonautes highlight their revolutionary potential in driving forward advancements in molecular biology and genomic research.

In conclusion, the application of Ago in nucleic acid biosensing is still in its early stages, and further exploration of the molecular and functional mechanisms of Ago is required. This includes the discovery of new Ago proteins, new nucleic acid enzymatic activities, and new physiological functions. These insights will provide theoretical guidance for the development of next-generation multi-target virus detection technologies and applications in the POCT field.

## Figures and Tables

**Table 1:** Characteristics of catalytically active prokaryotic Argonaute proteins.

Protein Name	Host	Guide ^a^	Target	Guide-Independent Activity	Iron	Reaction ^b^ Temperature	Reference
BlAgo	*Brevibacillus laterosporus*	P-DNA, OH-DNA	DNA	/	Mg^2+^, Mn^2+^	65 °C	[29]
CbAgo	*Clostridium butyricum*	P-DNA, OH-DNA	DNA	Nicking of Plasmid,chopping of dsDNA	Mg^2+^, Mn^2+^, Co^2+^	30–54 °C	[21,30]
CdAgo	*Clostridium disporicum*	P-DNA, OH-DNA	DNA	chopping of dsDNA, plasmid	Mn^2+^, weekly Mg^2+^, Co^2+^	37–55 °C	[31]
CpAgo	*Clostridium perfringens*	P-DNA, OH-DNA	DNA	/	Mg^2+^, Mn^2+^	37–50 °C	[32]
IbAgo	*Intestinibacter bartlettii*	P-DNA, OH-DNA	DNA	/	Mg^2+^, Mn^2+^	37–70 °C	[32]
KmAgo	*Kurthia massiliensis*	P-DNA, OH-DNA, P-RNA	RNA	Nicking of Plasmid	Mn^2+^, Mg^2+^, weakly Co^2+^	45–55 °C	[33]
LrAgo	*Limnothrix rosea*	P-DNA, OH-DNA	DNA	Nicking of Plasmid,chopping of dsDNA	Mn^2+^, Mg^2+^, weakly Co^2+^	50–54 °C	[21]
MbpAgo	*Mucilaginibacter paludis*	P-DNA, OH-DNA	RNA	/	Mn^2+^, Mg^2+^	30–55 °C	[34]
MfAgo	*Methanocaldococcus fervens*	P-DNA, OH-DNA, P-RNA	DNA	/	Mn^2+^, Mg^2+^, Co^2+^	80–90 °C	[35]
MjAgo	*Methanocaldococcus jannaschii*	P-DNA, OH-DNA	DNA	chopping of dsDNA, plasmid	Mg^2+^	85–95 °C	[36]
MpAgo	*Marinitoga piezophila*	OH-RNA, OH-DNA, P-DNA	DNA, RNA	/	Mn^2+^, Mg^2+^, weakly Ni^2+^	60 °C	[28]
PfAgo	*Pyrococcus furiosus*	P-DNA	DNA,	Nicking of Plasmid	Mn^2+^, Co^2+^	90–99.9 °C	[22,37]
Tce Ago	*Thermobrachium celere*	P-DNA, OH-DNA	DNA	/	Mn^2+^, Mg^2+^, Co^2+^, weekly Ca^2+^	40–60 °C	[38]
TtAgo	*Thermus thermophilus*	P-DNA	DNA, RNA	chopping of dsDNA	Mn^2+^, Mg^2+^	50–75 °C	[25,39]

^a^ Characteristics of the 5′-end of the guide: P—phosphate group, and OH—hydroxyl group; ^b^ the temperature at which Argonaute protein could cleave target.
